# Evaluation of Shear Bond Strengths of 3D Printed Materials for Permanent Restorations with Different Surface Treatments

**DOI:** 10.3390/polym16131838

**Published:** 2024-06-27

**Authors:** Mijoo Kim, Jimin Lee, Chan Park, Deukwon Jo, Bo Yu, Shahed Al Khalifah, Marc Hayashi, Reuben H. Kim

**Affiliations:** 1 Restorative Materials and Applied Dental Research Laboratory, UCLA School of Dentistry, Los Angeles, CA 90095, USA; jlee67@g.ucla.edu (J.L.); upgradepc@jnu.ac.kr (C.P.); deukwonjo@dentistry.ucla.edu (D.J.); boyu@dentistry.ucla.edu (B.Y.); salkhalifah@dentistry.ucla.edu (S.A.K.); mhayashi@dentistry.ucla.edu (M.H.); 2 Section of Restorative Dentistry, UCLA School of Dentistry, Los Angeles, CA 90095, USA; 3 Department of Prosthodontics, School of Dentistry, Dental Science Research Institute, Chonnam National University, Gwangju 500-757, Republic of Korea

**Keywords:** 3D printing, bonding strength, composite resin, DLP printer, silane, surface treatment, zirconia primer

## Abstract

The development of high-filled 3D printing resin necessitates a bonding protocol for dental indirect restorations to achieve optimal bond strength after cementation. This study evaluates shear bond strengths of high-filler 3D printed materials for permanent restorations with various surface treatments. Rodin Sculpture 1.0 (50% lithium disilicate fillers) and 2.0 Ceramic Nanohybrid (>60% zirconia and lithium disilicate fillers) were tested, with Aelite All-Purpose Body composite resin as control. Samples were prepared, post-cured, and sandblasted with alumina (25 µm). Surface roughness was analyzed using an optical profilometer. Two bonding protocols were compared. First, groups were treated with lithium disilicate silane (Porcelain Primer) or zirconia primer (Z-Prime Plus) or left untreated without a bonding agent. Beam-shaped resin cement (DuoLink Universal) specimens were bonded and stored in a 37 °C water bath. Second, additional sets of materials were coated with a bonding agent (All-Bond Universal), either followed by silane application or left untreated. These sets were then similarly stored alongside resin cement specimens. Shear bond tests were performed after 24 h. SEM images were taken after debonding. One-Way ANOVA and post hoc Duncan were performed for the statistical analysis. Rodin 1.0 exhibited increased adhesive failure with silane or zirconia primer coating, but significantly improved bond strengths with bonding agent application. Rodin 2.0 showed consistent bond strengths regardless of bonding agent application, but cohesive failure rates increased with bonding agent and filler coating. In all groups, except for Rodin 1.0 without bonding agent, silane coating increased cohesive failure rate. In conclusion, optimal shear bond strength for high-filler 3D printing materials can be achieved with silane coating and bonding agent application.

## 1. Introduction

The application of 3D printing technology in dentistry has expanded significantly, encompassing various uses such as dental casts, surgical guides, splints, aligners, and temporary restorations [1]. Its rise in popularity across industries is attributed to its procedural efficiency and environmental advantages compared to subtractive production methods [2,3,4]. The integration of supportive devices like oral and model scanners, along with advanced design software, has facilitated precise diagnosis and realization of dental professionals’ requirements. However, these applications typically require less precision compared to definitive restorations and allow room for lab or chairside adjustments. Researchers are leveraging the advantages of 3D printing technology to expand its applications to single crowns, long-span fixed prostheses, or removable partial/complete dentures, which demand higher mechanical requirements under loading and dimensional precision [5,6,7,8,9]. Ensuring the longevity and clinical success of dental restorations made with 3D printing technology necessitates the development of dental materials comparable to traditional cast metal or milled restorations.

Digital light processing (DLP) printers, wielding light-cured resins fortified with ceramic fillers, are revolutionizing 3D dental technology. Praised for their versatility, user-friendliness, precision, rapid production, and cost-efficiency, these printers have become indispensable in dental applications. However, the success of DLP printing hinges on the properties of the curable resin—specifically its strength, surface hardness, shrinkage, and water absorption—which are heavily influenced by the type, size, and content of the filler material [10,11]. While 3D printing materials formulated for splints, casts, or temporary restorations excel in these applications, their lower filler content can lead to vulnerabilities like wear, gloss reduction during occlusion, and susceptibility to impact or fatigue loading, especially when used for fixed prostheses.

To overcome these challenges associated with 3D printed fixed prostheses, recent advancements have led to the development of specialized materials tailored for permanent dental restorations. These materials exhibit a higher filler content than those intended for temporary or alternative applications. While marketed as suitable for permanent use, variations exist in their mechanical and biological properties, particularly in terms of filler type, size, and weight percentage. For definitive 3D printed restorations, stringent criteria such as strength, color stability, durability, and long-term biocompatibility are essential to ensure their clinical longevity, similar to conventional prosthetic materials. Additionally, managing viscosity and mitigating phase separation pose challenges, especially in formulations with high filler concentrations [12].

A previous study characterizing various 3D printing materials found that those formulated for permanent crowns (Ceramic Crown by SprintRay, Los Angeles, CA, USA, containing 50 wt% filler), dentures (OnX by SprintRay with 38 wt% filler and OnX Tough by SprintRay with 34 wt% filler), and temporary restorations (C&B MFH by NextDent, Soesterberg, Netherlands, with 3 wt% filler) displayed notably lower flexural strength, microhardness, and elastic modulus compared to direct composite resin (Filtek Supreme by 3M, St. Paul, MN, USA, with 72 wt% filler), milled resin blocks (with 72 wt% filler), and milled ceramics (IPS e.max CAD by Ivoclar Vivadent, Schaan, Liechtenstein). The study also revealed a positive linear relationship between filler content and modulus and hardness [11]. In another study by Karaoğlanoğlu et al., surface roughness, microhardness, and discoloration of resin-based CAD/CAM blocks (Cerasmart 270 and Grandio Blocs) were compared with 3D-printed permanent restorative resins (Crowntec and Permanent Crown). Both 3D printing resins, characterized by glass fillers with a particle size of 0.7 μm and a content of 30–50% by mass, exhibited similar surface roughness but displayed more discoloration and lower microhardness than CAD/CAM resin blocks [13]. It is worth noting that, depending on the materials used, some may be more prone to color change beyond clinically acceptable levels, despite being intended for permanent restorations, due to comparatively low filler content [14].

Recent advancements in 3D printing technology have spurred the development of resins with high ceramic fillers, exceeding 50% of lithium disilicate or zirconia. This innovation aims to enhance the mechanical performance of permanent dental restorations. A crucial aspect of these highly filled restorations is achieving robust bonding strength, in tandem with the inherent material strength. Any instability or partial debonding between the restoration and tooth substrate can lead to restoration or tooth fracture, marginal leakage, secondary caries, and periodontal issues during mastication or parafunctional movements, compromising overall integrity [15]. 

Traditionally, researchers have explored various surface treatment methods to enhance bonding, such as sandblasting, etching, silane coating, and bonding resins—techniques commonly employed with milled resin blocks and metal/ceramic restorations [16,17,18,19]. However, there is no consensus on standardized bonding protocols for permanent restorations fabricated with high-filler 3D printing resins. Additionally, the influence of different filler types (lithium disilicate, zirconia, or combinations) on bonding strength to resin cement remains largely unexplored. Therefore, this study aims to compare the shear bond strength of 3D printing materials intended for permanent restorations with various surface treatments.

## 2. Materials and Methods

### 2.1. Sample Preparation

Rodin Sculpture 1.0 Ceramic Nanohybrid (Rodin 1.0; with 50% lithium disilicate fillers) and 2.0 Ceramic Nanohybrid (Rodni 2.0; zirconia-infused nanohybrid resin, with over 60% filler content; Pac-Dent, Brea, CA, USA) were used for the experimental groups, with composite resin (Aelite All-Purpose Body, A1 shade with 0.4 to 0.7 µm in size and 73 wt% fillers; Bisco, Schaumberg, IL, USA) serving as the control group. Material specifications are detailed in Table 1. Each material was shaped into a disc (diameter 10 mm, thickness 2 mm) using a light curing unit (1300 mW/cm^2^, DeepCure; 3M ESPE, St. Paul, MN, USA) with overlapping curing following ISO 4049 standards [20] (n = 15/group). They were then post-cured in a curing box (LC-3DPrint Box; NextDent, Soesterberg, The Netherlands) for 20 min. Finally, all samples were embedded into acrylic resin (monomer and polymer resin; Great Lakes Orthodontics, Tonawanda, NY, USA) using a mold for the shear bond tester. All samples underwent sandblasting with alumina (25 µm) at 20 cm for 5 s under 2 bars.

### 2.2. Optical Profilometer Measurement

Prior to the bonding procedures, three types of specimens (composite resin, Rodin 1.0, and Rodin 2.0) were analyzed using an optical profilometer (NV-F2700, Nano System, Daejeon, Republic of Korea), which had a measurement resolution of 0.5 nm along the *Z* axis and 0.64 µm for the *X*–*Y* axis. Measurement was performed under WSI mode. The roughness of each specimen was calculated from the measurements and expressed as Sa, the arithmetic average of the 3D roughness surface profile.

### 2.3. Bonding Procedure

Initially, samples were treated with either silane (Porcelain Primer; Bisco, Schaumberg, IL, USA) or zirconia primer (Z-Prime Plus; Bisco, Schaumberg, IL, USA) or left untreated. Following drying, a bonding clamp with a mold insert (bonding surface diameter of 2.38 ± 0.03 mm) for shear bond strength was placed onto a 3D printed or composite resin disc embedded in acrylic resin. Adhesive resin cement, DuoLink Universal (Bisco, Schaumberg, IL, USA), was applied and light-cured for 20 s. Subsequently, the samples were stored in a 37 °C water bath for 24 h.

In the next step, All-Bond Universal (Bisco, Schaumberg, IL, USA) was applied to the specimens of three groups. They were air-dried for 20 s for the solvent to evaporate and light-cured for 20 s. Subsequently, surfaces were either coated with silane (Porcelain Primer) or left untreated. DuoLink Universal was applied following the same procedures as the previous step. These samples were also stored in the same conditions.

### 2.4. Shear Bond Test

After 24 h of cement setting, samples underwent testing using an Ultratester (Ultradent, South Jordan, UT, USA) at a crosshead speed of 1 mm/min. Bonding values were recorded in MPa units. Subsequently, the failure modes were analyzed using an optical microscope at a magnification of 30× following the shear bond tests. Three types of fracture patterns (adhesive, cohesive, or mixed failures) were recorded.

### 2.5. Scanning Electron Microscope (SEM) Imaging

After the shear bond tests, the debonded specimens were mounted on aluminum stubs, sputter-coated, and examined using a SEM (JEOL 5600 LVj; JEOL Ltd., Tokyo, Japan). To enhance electrical conductivity, a 10 nm thick gold layer was deposited onto the specimens using a sputter coater (BAL-TEC SCD 005 Sputter Coater; Balzers, Liechtenstein). An accelerating voltage of 5 kV was employed for imaging. Images were acquired in triplicate for each group, focusing on specific areas of interest at various magnifications.

### 2.6. Statistical Analyses

A Two-Way ANOVA (SPSS 26.0) with a Tukey post hoc test was employed to analyze the statistical differences among the groups. A significance level of α = 0.05 was utilized for all analyses.

## 3. Results

**Figure 1:**
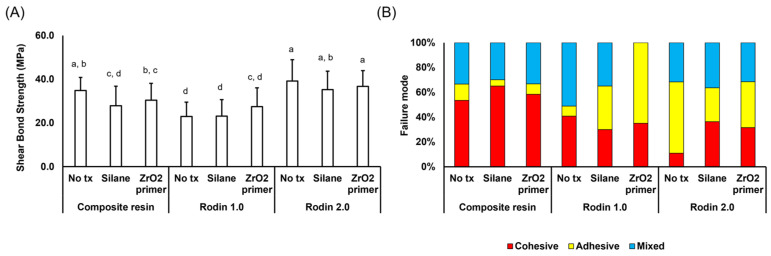
Shear bond strengths and failure modes of 3D printing materials and composite resin discs without bonding agent application. (**A**) Shear bond strength with different surface treatments. (**B**) Failure modes of the debonded samples. Different alphabets indicate significant differences between groups (*p* < 0.05).

Figure 1 presents the shear bond strengths and failure modes with either silane or zirconia primer. The Rodin 2.0 group exhibited significantly higher bond strengths in all conditions (*p* < 0.05). In the Rodin 2.0 group, the application of silane or zirconia primer reduced the occurrence of cohesive failure compared to the untreated group. The composite resin group displayed higher rates of cohesive failures across all groups compared to the Rodin 1.0 and 2.0 groups. Following the application of silane or zirconia primer, Rodin 1.0 showed a more pronounced adhesive failure, particularly with zirconia primer. Bond strengths showed a significant difference based on the material type (*p* < 0.05), while surface treatments did not have a notable impact (*p* > 0.05).

**Figure 2:**
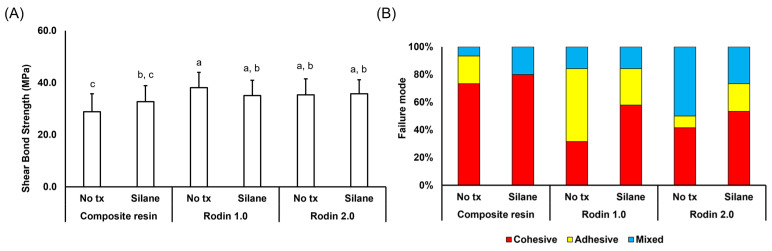
Shear bond strengths and failure modes of 3D printing materials and composite resin discs without bonding agent application. (**A**) Shear bond strength with or without silane coating. (**B**) Failure modes of the debonded samples. Different alphabets indicate significant differences between groups (*p* < 0.05).

After the bonding agent treatment, Rodin 1.0 significantly increased its bond strength compared to the untreated samples (22.93 ± 6.57 and 23.16 ± 7.48 to 38.12 ± 5.89 and 35.08 ± 5.87, respectively) in Figure 2A. Rodin 1.0 and 2.0 groups did not exhibit significant differences across all conditions (*p* > 0.05) and had significantly higher bond strengths than the control group (*p* < 0.05). The application of a bonding resin did not alter the bonding strengths following silane treatment. However, a reduction in adhesive failures and an increase in cohesive failures were observed with the three test materials after silane coating in Figure 2B. In particular, the composite resin group showed no adhesive failures after silane coating. Bond strengths with the application of the bonding agent were significantly affected only by the material type (*p* < 0.05).

**Figure 3:**
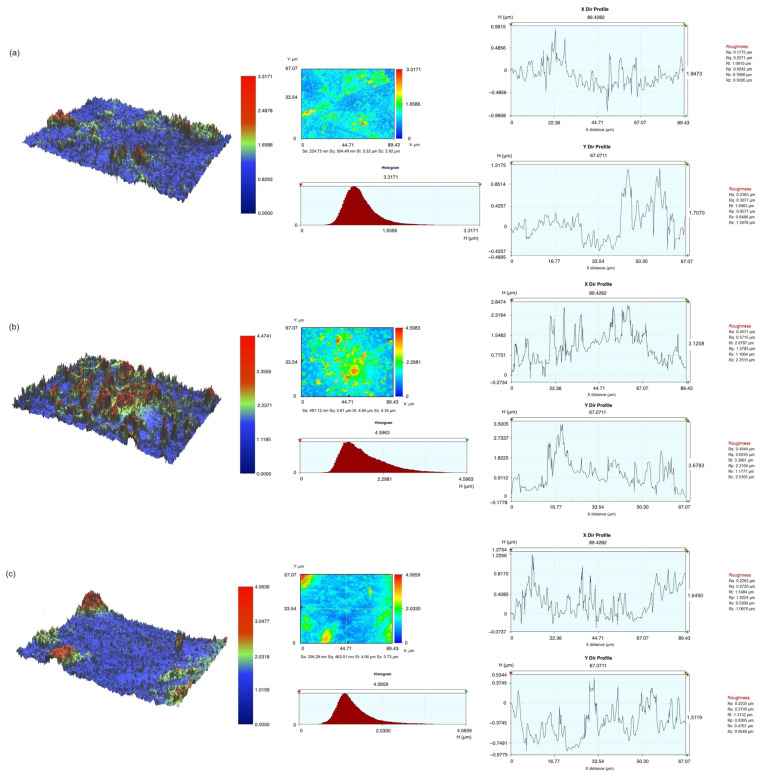
Optical profilometer measurement before bonding procedures. (**a**) Composite resin, (**b**) Rodin 1.0, (**c**) Rodin 2.0.

The representative surface topography of three materials after sandblasting treatment is shown in Figure 3. The Sa values were 224.73 nm, 481.12 nm, and 336.29 nm, respectively. Additionally, color images indicated a rougher surface for Rodin 1.0 and Rodin 2.0 compared to the composite resin group after alumina sandblasting.

**Figure 4:**
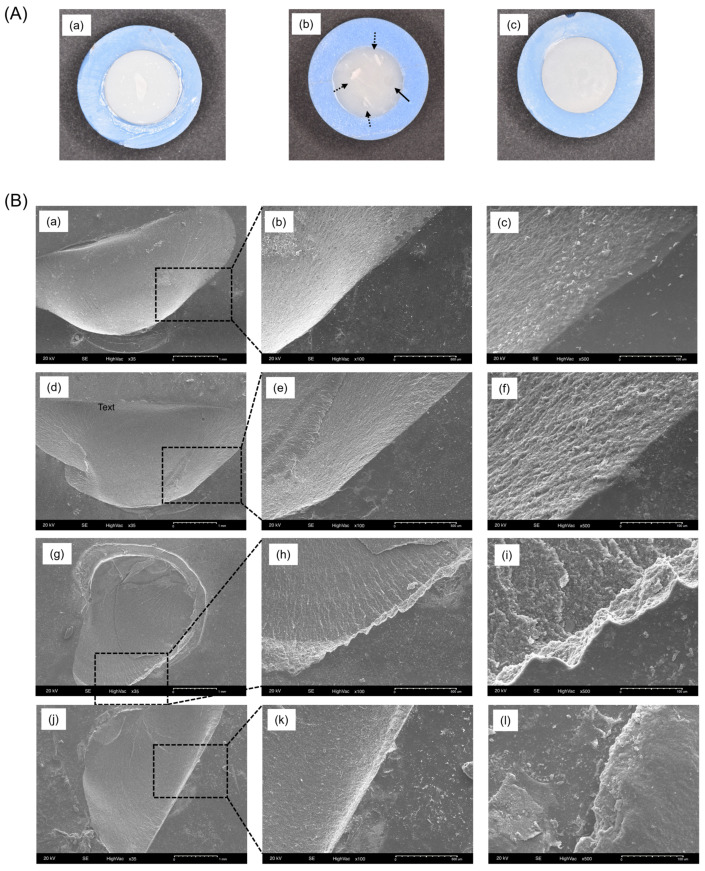
Surface images after debonding. (**A**) Representative photos of debonded specimens. (**A-a**) composite resin; cohesive failure, (**A-b**) Rodin 1.0; 3 out of 4-mixed (dash type arrow), 1/4-adhesive failure (solid line arrow), (**A-c**) Rodin 2.0; adhesive failure. (**B**) SEM images of debonded specimens. (**a**,**d**,**g**,**j**) 35×, (**b**,**e**,**h**,**k**) 100×, (**c**,**f**,**i**,**l**) 500× magnifications. (**B-(a–c)**) Composite resin; cohesive failure, (**B-(d–f)**) Rodin 1.0; cohesive failure, (**B-(g–i)**) Rodin 1.0; mixed failure, (**B-(j–l)**) Rodin 2.0; cohesive failure.

Figure 4 illustrates representative photo and SEM images after obtaining bonding data. Figure 4A-a displays the composite resin group with cohesive failure, which was the most common failure mode seen in that group. Figure 4A-b depicts different failure modes in Rodin 1.0 sample, with three out of four showing mixed failure and one showing adhesive failure, while Figure 4A-c shows the Rodin 2.0 group with adhesive failure. SEM images demonstrate magnified fracture modes in Figure 4B. The composite resin group (Figure 4B-(a–c)) displays a smooth and clean border-lined surface after cohesive fracture, contrasting with the surfaces of the Rodin 1.0 and 2.0 groups.

## 4. Discussion

Three-dimensional printing technology has the potential to revolutionize dental restorations, offering reduced fabrication time, simplified procedural steps, and increased access to underserved areas. A recent shift in US dental insurance policies further highlights this potential. Effective 1 January 2023, CDT (Code on Nomenclature) coding now allows for reimbursement of 3D printed resin crowns with filler content exceeding 50% as permanent restorations, placing them alongside established options like porcelain-fused-to-metal (PFM) and zirconia restorations. However, widespread clinical adoption necessitates a thorough understanding of the mechanical, physical, chemical, and biological properties of these printed materials. This study specifically focuses on the bonding efficacy of these resins compared to conventional high-filler composites. By evaluating their bonding strength and durability, we aim to assess their suitability for lasting dental applications.

Aluminum oxide sandblasting created a complex three-dimensional rough surface on the specimens, with varying peak and valley heights and self-similar fractal properties [21]. This process resulted in rougher surfaces for Rodin 1.0 and 2.0, likely because the composite resin used in these materials has a higher filler content compared to the 3D printing resin, making it more resistant to sandblasting. In the 3D printed resin samples, the sandblasting eroded the resin matrix between the ceramic fillers, creating the observed deeper grooves. To determine the appropriate intensity and duration of such micro-mechanical surface treatments for future studies, it is crucial to analyze how these factors impact potential damage to 3D printing resins [22].

Figure 1 and Figure 2 clearly demonstrate the enhanced bonding strength achieved by applying a bonding agent, particularly in the Rodin 1.0 group. While bonding agent application did not significantly impact bonding strength for Rodin 2.0 and composite resin groups, it did decrease adhesive failures (58% to 8.3% for non-treated; 27% to 20% for silane) and significantly increase cohesive failures (11.0% to 41.6% for non-treated; 36% to 53.3% for silane). Bonding agents play a crucial role in ensuring a strong connection between a 3D-printed resin substrate and subsequent resin materials after sandblasting [23,24,25]. They achieve this primarily by two methods: reducing the substrate’s surface tension for better wetting [26,27,28] and forming direct chemical bonds. In particular, universal adhesives were proved to outperform two- or three-step bonding agents [29]. Moreover, many universal adhesives, including All-Bond Universal, contain 10-Methacryloyloxydecyl dihydrogen phosphate (10-MDP) to chemically bond with diverse surfaces, including zirconia fillers in composites [25,30,31]. Thus, current findings provide strong evidence supporting the use of bonding agents when cementing 3D printed resin materials. 

Silane and zirconia primer primarily influenced the observed failure modes, not necessarily immediate bonding strength. Traditionally, silane, applied after hydrofluoric acid etching, chemically bonds ceramics like lithium disilicate to subsequent materials. Karabekiroglu et al. suggests silane may improve long-term bond strength for repairs while immediate or short-term benefits are less clear as seen in this study [32]. Similarly, zirconia primer, designed for zirconia restorations which are difficult to etch, uses 10-MDP for enhanced chemical bonding [33,34]. In this study, silane and zirconia primer did not significantly impact initial bonding strength, which aligns with research focusing on their influence on failure modes, not immediate strength [25,35,36]. Additionally, Rodin 2.0, containing a smaller proportion of zirconia compared to lithium disilicate (information provided by a manufacturer), was not significantly affected by the zirconia primer, which accounts for the similar failure modes either with silane or zirconia primer observed in Figure 1.

Ceramic coatings do not appear to function effectively even in the absence of bonding agent application, particularly for Rodin 1.0. As illustrated in Figure 1, the proportions of cohesive and mixed failure rates for Rodin 1.0 decreased even after surface treatment (92.6% for non-treated; 80.0% for silane; 35% for zirconia primer), while maintaining nearly identical levels of cohesive failure rates before and after surface treatments. The interaction of Si with inorganic fillers results in the formation of a thick and multi-phase interfacial layer between the composite resin and the adhesive, hindering direct interaction between the methacrylate monomers from the adhesive and the polymer network of the already polymerized composite resin [37,38]. The current study is consistent with previous studies indicating minimal improvement in bond strength with silane treatment [36,39,40]. Additionally, Rodin Sculpture 1.0 comprises a resin matrix and fillers in nearly equal ratios, suggesting that bonding to the matrix is as crucial for bonding strength as bonding to ceramic fillers, necessitating bonding agent coating before filler coating.

Enhancing micromechanical retention through processes like sandblasting or etching is vital for achieving optimal resin bonding, alongside chemical bonding. Resins with higher filler content tend to develop increased surface roughness primarily between each filler particle, as the filler material remains intact during sandblasting. Loomans et al. illustrated the diverse surface roughness resulting from various etching techniques applied to hybrid-filled and nano-filled composite resins. Depending on the protocols and resin type, both exhibited notably distinct surface textures [41]. Junior et al. observed unique surface patterns following sandblasting in nanofill and microhybrid composite resins with filler contents of around 60% [42]. Variations in filler content, size, and shape can generate diverse micromechanical patterns through both mechanical and chemical mechanisms. As a result, Rodin 1.0 and 2.0, each possessing distinct filler content, exhibited contrasting surface roughness profiles, as evident from the optical profilometry visualization. These disparities could significantly affect bonding strengths, particularly in scenarios lacking appropriate chemical bonding due to the absence of bonding agent application. 

While this study offers valuable insights into new materials, several limitations must be acknowledged. First, the 3D printed materials utilized were not cured within the printer, potentially impacting their inherent properties and surface homogeneity. Additionally, post-curing processes, influenced by device variations and surrounding gas environments, may further affect the final product’s degree of conversion [43,44]. Future investigations will employ 3D printer-specific curing methods to provide a more clinically relevant understanding of material behavior. Second, the study only included one type of each essential component (resin cement, silane, or zirconia primer), limiting the generalizability of the findings. Conducting broader comparisons across different materials is essential for a comprehensive understanding of 3D-printed material cementation. Third, specific compositions for the materials were not provided by the manufacturer, which hindered a deeper analysis of their bonding mechanisms. Further exploration through component and chemical bond analyses would be beneficial in elucidating their behavioral mechanisms. Finally, future research endeavors will incorporate a more clinically relevant model, including indirect restoration designs and simulated chewing forces, to better evaluate bonding efficacy under realistic conditions.

## 5. Conclusions

Considering the limitations of the current study, it is recommended to utilize bonding agent and silane primer coating for achieving optimal bond strength when cementing restorations made from 3D printing resin used in this study. Further investigations involving restoration contours and chewing simulation with various types of 3D printing materials for permanent restorations are warranted to provide comprehensive support for the clinical utilization of dental 3D technology.

## Figures and Tables

**Table 1:** Materials used in this study.

Materials	Shade	Manufacturer	Lot No.	Compositions
Rodin Sculpture 1.0 Ceramic Nanohybrid	A1	Pac-Dent	309002	Methyl methacrylate resin, Photo initiator, Photo inhibitor, Pigment, Ceramic fillers
Rodin Sculpture 2.0 Ceramic Nanohybrid	A1	Pac-Dent	312152	Monomer, Oligomer, Photo initiator, Photo inhibitor, Pigment, Ceramic fillers
Aelite All-Purpose Body	A1	Bisco	2100007080	Ethoxylated bisphenol A dimethacrylate, Triethyleneglycol dimethacrylate, Glass fillers, Amorphous silica
Porcelain Primer		Bisco	2300001686	Acetone, 3-(Trimethoxysilyl)propyl-2-Methyl-2-Propenoic Acid (3-MPTS), Acetic acid
Z-Prime Plus		Bisco	2100003987	BisGMA, 2-Hydroxyethyl Methacrylate (HEMA), 10-Methacryloyloxydecyl Dihydrogen Phosphate (10-MDP)
All-Bond Universal		Bisco	220001298	BisGMA, 2-Hydroxyethyl Methacrylate, 10-Methacryloyloxydecyl Dihydrogen Phosphate, Ethyl 4-dimethylaminobenzoate

## Data Availability

The data presented in this study are openly available in FigShare at https://doi.org/10.6084/m9.figshare.25864450.v1 (accessed on 20 May 2024).
